# Superiority of quadratic over conventional neural networks for classification of gaussian mixture data

**DOI:** 10.1186/s42492-022-00118-z

**Published:** 2022-09-28

**Authors:** Tianrui Qi, Ge Wang

**Affiliations:** Department of Computer Science, Rensselaer Polytechnic Institute, NY 12180 Troy, USA

**Keywords:** Artificial neural networks, Quadratic neurons, Quadratic neural networks, Backpropagation, Classification, Gaussian mixture models

## Abstract

To enrich the diversity of artificial neurons, a type of quadratic neurons was proposed previously, where the inner product of inputs and weights is replaced by a quadratic operation. In this paper, we demonstrate the superiority of such quadratic neurons over conventional counterparts. For this purpose, we train such quadratic neural networks using an adapted backpropagation algorithm and perform a systematic comparison between quadratic and conventional neural networks for classificaiton of Gaussian mixture data, which is one of the most important machine learning tasks. Our results show that quadratic neural networks enjoy remarkably better efficacy and efficiency than conventional neural networks in this context, and potentially extendable to other relevant applications.

## Introduction

In machine learning, the mainstream approach is now artificial neural networks (ANNs), especially deep neural networks. Usually, a neural network consists of several layers of neurons, each of which consists of a linear compartment in the form of the inner product of inputs and weights and a nonlinear unit known as an activation function to make a signal on (activated) or off (attenuated). Deep neural networks have been recently shown to achieve remarkable successes in various applications such as natural language processing [1, 2], auto-driving [3–5], game-playing [6], image analysis [7, 8], and image reconstruction [9].

Classification/clustering is one of the essential pattern recognition techniques in machine learning, and has a wide arrange of applications such as bioinformatics [10, 11] and medial imaging [12, 13]. It is well known that the Gaussian mixture model (GMM) is the most popular data model. Since the prior probability of each Gaussian component is typically not given, known as latent variables, the correct parameters of GMM are solved using the expectation-maximization (EM) algorithm. Alternatively, a neural network approach can be used to classify GMM data. Clearly, the decision boundary for the classification can be viewed as a complicated function where a network with a large number of neurons can approximate that boundary. After the classification network is trained, the inference by the trained network is more efficient than the EM algorithm, which is iterative and time-consuming.

In our previous study [14–19], a new type of neurons, referred to as quadratic neurons, was introduced, where the inner product inside a conventional neuron is upgraded to a quadratic function. The initial motivation is to enrich the diversity of artificial neurons, inspired by the fact that the biological diversity exists at the cellular level, and such diversity enables efficiency, flexibility, functionality, and other benefits. Hence, it is hypothesized that a quadratic neural network would be advantageous similarly, which can, for example, approximate a given function with a lighter structure than a conventional neural network.

The main purpose of this paper is to highlight the superiority of quadratic over conventional neural networks with the classification task as an illustrative example. The rest of the paper is organized as follows. In the next section, we review the theoretical minimum error in the GMM classification and the EM algorithm that is traditionally used to reach that error bound. In the third section, we present our procedure for initializing and training the conventional and quadratic networks with an adapted backpropagation (BP) algorithm. In the fourth section, we perform numerical experiments systematically and establish the superiority of quadratic networks over the conventional counterparts in the GMM classification. Finally, in the last section we discuss relevant issues and conclude the paper.

## Methods

### GMM-based classification error

In statistical classification, the Bayes error rate is theoretically optimal. In practice, without knowing latent GMM parameters, the Bayes error rate cannot be directly calculated. To close the gap, the classic EM algorithm can be used to approximate the optimal error rate, which is the benchmark to evaluate the performance of classification neural networks.

#### Bayes error

Given the mean $\mu$, covariance $\mathbf {C}$, and prior probability $\pi$ of each Gaussian component of GMM $\mathcal {N}$, the posterior probability $p\left( z_{k}=1|x_n \right)$ is calculated by

1 $$\begin{aligned} p\left( z_{k}=1|x_{n}\right) =\frac{\pi _{k} \mathcal {N} \left( x_{n}|\mu _{k} ,\mathbf {C}_{k} \right) }{\sum \nolimits ^{K}_{i=1} \pi _{i} \mathcal {N} \left( x_{n}|\mu _{i} ,\mathbf {C}_{i} \right) } \end{aligned}$$

which means a *D* dimensional sample vector $x_n$, $n\in \left\{ 1,\dots ,N\right\}$, should be assigned to the $\hat{\mathbf {y}}x_{n}$ Gaussian component,

2 $$\begin{aligned} \hat{\mathbf {y}}_{n}=\arg \max _{k\in \left\{ 1,\dots ,K\right\} } p\left( z_{k}=1|x_{n}\right) \end{aligned}$$

where *D*, *N*, and *K* represent the dimensionality of the sample vector, the sample size and the number of Gaussian components respectively. We can obtain the Bayesian inference results, a size *N* vector $\hat{\mathbf {y}}$, by applying Eq. 2 to the entire sample pool $\mathbf {x} =\left[ x_{1},\dots ,x_{N}\right] ^{T}$ and compare it with the ground truth labels $\mathbf {t}$. However, in most of real cases all these GMM parameters are not directly known. Fortunately, we can use the EM algorithm to estimate them, as described in the following subsection.

Note that the inference $\hat{\mathbf {y}}$ cannot be directly used as the predicting label of each sample since our task is clustering instead of classification. For example, while the ground truth parameters are $\theta = \{\theta _{1}, \theta _{2}, \theta _{3}, \theta _{4}\}$, the results from the EM algorithm can be $\hat{\theta } = \{\hat{\theta }_{2}, \hat{\theta }_{4}, \hat{\theta }_{1}, \hat{\theta }_{3}\}$, $\hat{\theta _{k}}\approx \theta _{k}$ for $k\in \{1, 2, 3, 4\}$. Hence, we have to perform an order correction, i.e., rearranging $\hat{\theta }$ as $\{ \hat{\theta }_{1} ,\hat{\theta }_{2} ,\hat{\theta }_{3} ,\hat{\theta }_{4} \}$.

A solution to this problem is to perform an exclusive search so that the accuracy or loss can be optimized. By doing so, the best match will be found as our final result. More efficiently, the alternating variables method can be used as described in Algorithm 1, a common derivative-free method for numerical optimization, with the idea to maximize the accuracy by exchanging two coordinates each time and fixing all the remaining ones. We set the *MaxCycle* according to the number of Gaussian components *K*, and in our experiment $MaxCycle = 20$, which is sufficiently large for $K = 8$.

Then, we compute Eq. 2 using parameters after the order correction we present above for the Bayesian inference results $\mathbf {y}$ and gain the Bayes error as the banchmark of performance of neural networks. 
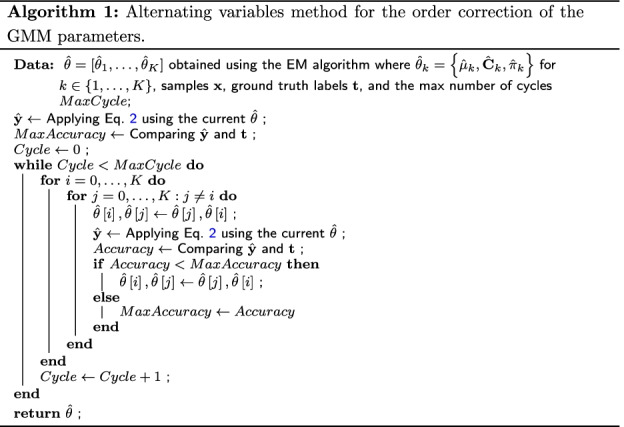


#### EM algorithm

As a classic iterative method, the EM algorithm consists of the following two steps: expectation (E) and maximization (M). The E step evaluates the expectation function based on the currently available intermediate parameters, and the M step updates the intermediate parameters to maximize the expectation function. To estimate all the $\mu , \mathbf {C}, \pi$ parameters, the expectation function in the E step is the posterior probability $p\left( z_{k}=1|\mathbf {x}\right)$ for $k = 1, \dots , K$.

To start the EM procedure, for $k = 1, \dots , K$, we initialize $\mu ^{\left( 0\right) }_{k}$ with a size *D* vector that filled with values from the standard normal distribution, $\mathbf {C}^{\left( 0\right) }_{k}$ with *D* by *D* identity matrix and $\pi ^{\left( 0\right) }_{k} =1/K$. Then, for the *j*th iteration, $j\ge 0$, the posterior probability in the E step is computed as

3 $$\begin{aligned} p^{\left( j\right) }\left( z_{k}=1|\mathbf {x} \right) =\frac{\pi ^{\left( j\right) }_{k} \mathcal {N} \left( \mathbf {x} |\mu ^{\left( j\right) }_{k} ,\mathbf {C}^{\left( j\right) }_{k} \right) }{\sum \nolimits ^{K}_{i=1} \pi ^{\left( j\right) }_{i} \mathcal {N} \left( \mathbf {x} |\mu ^{\left( j\right) }_{i} ,\mathbf {C}^{\left( j\right) }_{i} \right) } \end{aligned}$$

in terms of the current parameters $\mu ^{\left( j\right) }_{k} ,\mathbf {C}^{\left( j\right) }_{k} ,\pi ^{\left( j\right) }_{k}$, $k= 1, \dots , K$. After this E step, the M step goes as follows:

4 $$\begin{aligned} \begin{aligned} \mu ^{\left( j+1\right) }_{k}&=\frac{\sum \nolimits ^{N}_{n=1} p^{\left( j\right) }\left( z_{k}=1|x_{n}\right) x_{n}}{\sum \nolimits ^{N}_{n=1} p^{\left( j\right) }\left( z_{k}=1|x_{n}\right) }\\ \mathbf {C}^{\left( j+1\right) }_{k}&=\frac{\sum \nolimits ^{N}_{n=1} \left( x_{n}-\mu _{k} \right) p^{\left( j\right) }\left( z_{k}=1|x_{n}\right) \left( x_{n}-\mu _{k} \right) ^{T} }{\sum \nolimits ^{N}_{n=1} p^{\left( j\right) }\left( z_{k}=1|x_{n}\right) }\\ \pi ^{\left( j+1\right) }_{k}&=\frac{1}{N} \sum \limits ^{N}_{n=1} p^{\left( j\right) }\left( z_{k}=1|x_{n}\right) \end{aligned} \end{aligned}$$

The E and M steps are repeated until the parameters being estimated converge within a pre-specified range or a maximum number of iterations is finished. With these estimated GMM parameters, Eq. 2 and Algorithm 1 can be used for GMM-oriented classificaiton.

### Neural network training

**Fig. 1:**
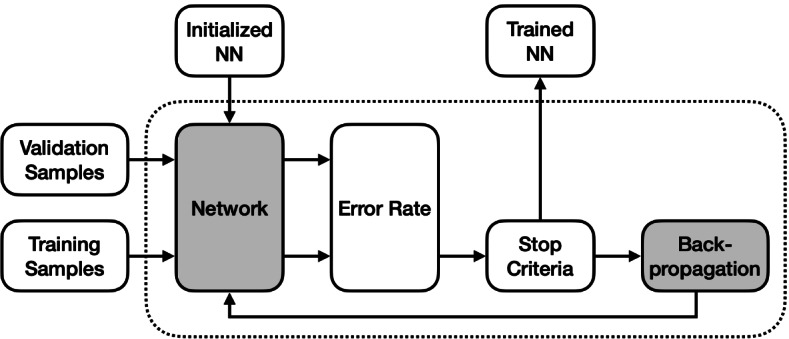
Neural network training as a computational optimization process with respect to an objective function which is the error rate for classification, without loss of generality

Training a neural network involves two steps: initialization which sets up network parameters appropriately, and optimization which adjusts the neural parameters iteratively. An optimizer used in the second step is illustrated in Fig. 1. The key idea is to perform computational optimization using the well-known BP algorithm with respect to an objective or loss function.

While the conventional and quadratic neural networks can be trained based on the same idea of computational optimization, they differ in specific steps, since the chain rule must be applied to different functions that summarize data (i.e., inter product versus quadratic operation). Specifically, let us formulate the forward and BP processes in the following two subsections respectively, and then describe the whole process in the third subsection.

#### Forward computation

**Fig. 2:**
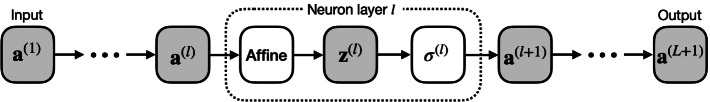
Forward computation of a feed-forward neural network with *L* layers of neurons

An exemplary feed-forward neural network is shown in Fig. 2, including input, hidden, and output layers. There are *L* layers in total, in each of which there is a number of neurons. A typical layer first implements affine transforms for conventional neurons and quadratic operations for quadratic neurons, and then nonlinear activations $\sigma ^{(l)}$ are performed, which are common for conventional and quadratic neurons.

**Fig. 3:**
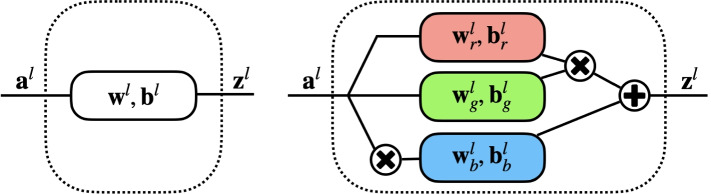
Illustration of the affine layer of conventioinal (left) and quadratic (right) neurons respectively

An illustration of the affine layer of conventioinal and quadratic neurons is shown in Fig. 3. For a conventional neural network, the affine transform can be expressed in terms of a input matrix $\mathbf {a}^{(l)}$ and a weight matrix $\mathbf {w}^{(l)}$ plus a bias row vector $\mathbf {b}^{(l)}$ as follows:

5 $$\begin{aligned} \mathbf {z}^{\left( l\right) } =\mathbf {a}^{\left( l\right) } \mathbf {w}^{\left( l\right) } +\mathbf {b}^{\left( l\right) } \end{aligned}$$

For a quadratic neural network, the quadratic transform can be expressed as

6 $$\begin{aligned} \begin{aligned} \mathbf {z}^{\left( l\right) } =&\left( \mathbf {a}^{\left( l\right) } \mathbf {w}^{\left( l\right) }_{r} +\mathbf {b}^{\left( l\right) }_{r} \right) \circ \left( \mathbf {a}^{\left( l\right) } \mathbf {w}^{\left( l\right) }_{g} +\mathbf {b}^{\left( l\right) }_{g} \right) \\&\qquad +\left( \mathbf {a}^{\left( l\right) } \circ \mathbf {a}^{\left( l\right) } \right) \mathbf {w}^{\left( l\right) }_{b} +\mathbf {c}^{\left( l\right) } \end{aligned} \end{aligned}$$

where $\mathbf {a}^{\left( l\right) } \mathbf {w}^{\left( l\right) }$ stands for matrix multiplication and $\circ$ means an element-wise square operation. In this study, the ReLU function is used as the activation function, but if the *l*-th layer is the last layer of the network, i.e., $l = L$, the softmax function is computed instead. Therefore, the output of each layer is computed as follows:

7 $$\begin{aligned} \mathbf {a}^{\left( l+1\right) } =\sigma ^{\left( l\right) } \left( \mathbf {z}^{\left( l\right) } \right) = \left\{ \begin{array}{ll} \max \left( \mathbf {z}^{\left( l\right) } ,0\right) &{}l\ne L\\ e^{\mathbf {z}^{\left( l\right) } }/\sum e^{\mathbf {z}^{\left( l\right) } }&{}l=L \end{array}\right. \end{aligned}$$

In other words, the input to the forward process is a *N* by *D* sample matrix $\mathbf {a}^{(1)} = \mathbf {x}$, and output is a *N* by *K* matrix $\mathbf {a}^{(L+1)}$. The prediction of each sample vector $x_n$ is quantified by

8 $$\begin{aligned} \mathbf {y}_{n}=\arg \max _{k\in \left\{ 1,\cdots ,K\right\} } \mathbf {a}^{\left( L+1\right) }_{n,k} \end{aligned}$$

The loss or error is produced when the prediction differs from the ground truth. Note that in the forward computation we compute and store the output of each affine transform, which are subsequently used for the gradient descent search in the BP process described in the following subsection.

#### BP formulation

**Fig. 4:**
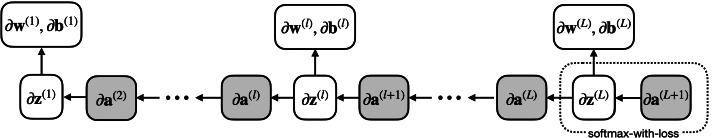
BP process to train a neural network of *L* layers

To optimize a neural network, we perform numerical optimization. Specifically, we first find the partial derivatives with respect to each of the parameters and update them via gradient descent search at a suitable step size (learning rate). Using the chain rule, this process was formulated as the well-known BP algorithm, which is widely used to train a neuronal network. As its name indicates, the BP process computes the partial derivatives layer-wise from the output layer to the input layer. A brief BP diagram is shown in Fig. 4.

Let *Q* stand for the cross-entropy loss value defined as

$$\begin{aligned} Q=-\sum \limits ^{N}_{n=1} \mathbf {t}_{n} \log \mathbf {y}_{n} \end{aligned}$$

where *N* is the number of sample vectors, $\mathbf {y}_n$ is the predicted result, and $\mathbf {t}_n$ is the ground truth label for each of the samples $x_n$. Recall that the activation function of the output layer is the softmax function, hence the gradient of the output layer can be computed as

9 $$\begin{aligned} \frac{\partial Q}{\partial \mathbf {z}^{\left( L\right) } } =\mathbf {y} -\mathbf {t} \end{aligned}$$

If $l\ne L$, the activation function is the ReLU function, and we have

10 $$\begin{aligned} \begin{aligned} \frac{\partial Q}{\partial \mathbf {z}^{\left( l\right) } }&=\frac{\partial Q}{\partial \mathbf {a}^{\left( l+1\right) } } \frac{\partial \mathbf {a}^{\left( l+1\right) } }{\partial \mathbf {z}^{\left( l\right) } } \\&=\left\{ \begin{array}{ll} {\partial Q/\partial \mathbf {a}^{\left( l+1\right) }} &{} \mathbf {a}^{\left( l+1\right) } >0\\ 0 &{} \mathbf {a}^{\left( l+1\right) } \le 0 \end{array}\right. ,\qquad l\ne L \end{aligned} \end{aligned}$$

For a conventional neural network, we know that

11 $$\begin{aligned} \begin{aligned} \frac{\partial Q}{\partial \mathbf {w}^{\left( l\right) }}&= \frac{\partial Q}{\partial \mathbf {z}^{\left( l\right) }} \frac{\partial \mathbf {z}^{\left( l\right) }}{\partial \mathbf {w}^{\left( l\right) }}=\left( \mathbf {a}^{\left( l\right) } \right) ^{T} \frac{\partial Q}{\partial \mathbf {z}^{\left( l\right) }}\\ \frac{\partial Q}{\partial \mathbf {b}^{\left( l\right) }}&=\frac{\partial Q}{\partial \mathbf {z}^{\left( l\right) } } \frac{\partial \mathbf {z}^{\left( l\right) } }{\partial \mathbf {b}^{\left( l\right) } } =\sum \frac{\partial Q}{\partial \mathbf {z}^{\left( l\right) }} \end{aligned} \end{aligned}$$

where

12 $$\begin{aligned} \frac{\partial Q}{\partial \mathbf {a}^{\left( l\right) }} = \frac{\partial Q}{\partial \mathbf {z}^{\left( l\right) } } \frac{\partial \mathbf {z}^{\left( l\right) }}{\partial \mathbf {a}^{\left( l\right) }} = \frac{\partial Q}{\partial \mathbf {z}^{\left( l\right) } } \left( \mathbf {w}^{\left( l\right) } \right) ^{T} \end{aligned}$$

The same chain rule can be applied to optimize a quadratic neural network layer-wise. Specifically, let us consider Eq. 6 in the following three parts:

$$\begin{aligned} \begin{aligned} \mathbf {z}^{\left( l\right) }&= \underbrace{\left( \mathbf {a}^{\left( l\right) } {\mathbf {w} }^{\left( l\right) }_{r} +\mathbf {b}^{\left( l\right) }_{r} \right) }_{\mathbf {z}^{\left( l\right) }_{r} } \circ \underbrace{\left( \mathbf {a}^{\left( l\right) } {\mathbf {w} }^{\left( l\right) }_{g} +\mathbf {b}^{\left( l\right) }_{g} \right) }_{\mathbf {z}^{\left( l\right) }_{g}}\\&\qquad +\underbrace{\left( \mathbf {a}^{\left( l\right) } \circ \mathbf {a}^{\left( l\right) } \right) {\mathbf {w} }^{\left( l\right) }_{b} +\mathbf {c}^{\left( l\right) } }_{\mathbf {z}^{\left( l\right) }_{b} } \end{aligned} \end{aligned}$$

and we have

$$\begin{aligned} \begin{aligned} \frac{\partial Q}{\partial {\mathbf {z} }^{\left( l\right) }_{r}}&=\frac{\partial Q}{\partial {\mathbf {z} }^{\left( l\right) }} \frac{\partial {\mathbf {z} }^{\left( l\right) } }{\partial {\mathbf {z} }^{\left( l\right) }_{r}} = {\mathbf {z}}^{\left( l\right) }_{g} \frac{\partial Q}{\partial \mathbf {z}^{\left( l\right) }}\\ \frac{\partial Q}{\partial {\mathbf {z} }^{\left( l\right) }_{g}}&=\frac{\partial Q}{\partial {\mathbf {z}}^{\left( l\right) }} \frac{\partial {\mathbf {z}}^{\left( l\right) }}{\partial {\mathbf {z}}^{\left( l\right) }_{g}} ={\mathbf {z}}^{\left( l\right) }_{r} \frac{\partial Q}{\partial \mathbf {z}^{\left( l\right) }}\\ \frac{\partial Q}{\partial {\mathbf {z} }^{\left( l\right) }_{b}}&= \frac{\partial Q}{\partial {\mathbf {z} }^{\left( l\right) } } \frac{\partial {\mathbf {z} }^{\left( l\right) } }{\partial {\mathbf {z} }^{\left( l\right) }_{b} } =\frac{\partial Q}{\partial \mathbf {z}^{\left( l\right) }} \end{aligned} \end{aligned}$$

Then, the gradients with respect to the parameters in the three parts can be respectively found as follows:

13 $$\begin{aligned} \begin{aligned} \frac{\partial Q}{\partial \mathbf {w}^{\left( l\right) }_{r}}&=\frac{\partial Q}{\partial {\mathbf {z}}^{\left( l\right) }_{r}} \frac{\partial {\mathbf {z}}^{\left( l\right) }_{r}}{\partial \mathbf {w}^{\left( l\right) }_{r}} =\left( \mathbf {a}^{\left( l\right) } \right) ^{T} \frac{\partial Q}{\partial {\mathbf {z}}^{\left( l\right) }_{r}}\\ \frac{\partial Q}{\partial \mathbf {b}^{\left( l\right) }_{r} }&=\frac{\partial Q}{\partial {\mathbf {z} }^{\left( l\right) }_{r} } \frac{\partial {\mathbf {z} }^{\left( l\right) }_{r} }{\partial \mathbf {b}^{\left( l\right) }_{r} } =\sum \frac{\partial Q}{\partial {\mathbf {z}}^{\left( l\right) }_{r}}\\ \frac{\partial Q}{\partial \mathbf {w}^{\left( l\right) }_{g} }&=\frac{\partial Q}{\partial {\mathbf {z} }^{\left( l\right) }_{g} } \frac{\partial {\mathbf {z} }^{\left( l\right) }_{g} }{\partial \mathbf {w}^{\left( l\right) }_{g} } =\left( \mathbf {a}^{\left( l\right) }\right) ^{T} \frac{\partial Q}{\partial {\mathbf {z} }^{\left( l\right) }_{g}}\\ \frac{\partial Q}{\partial \mathbf {b}^{\left( l\right) }_{g} }&=\frac{\partial Q}{\partial {\mathbf {z} }^{\left( l\right) }_{r} } \frac{\partial {\mathbf {z} }^{\left( l\right) }_{r} }{\partial \mathbf {b}^{\left( l\right) }_{g} } =\sum \frac{\partial Q}{\partial {\mathbf {z} }^{\left( l\right) }_{g}}\\ \frac{\partial Q}{\partial \mathbf {w}^{\left( l\right) }_{b} }&=\frac{\partial Q}{\partial {\mathbf {z} }^{\left( l\right) }_{b} } \frac{\partial {\mathbf {z} }^{\left( l\right) }_{b} }{\partial \mathbf {w}^{\left( l\right) }_{b} } = \left( \mathbf {a}^{\left( l\right) } \circ \mathbf {a}^{\left( l\right) } \right) ^{T} \frac{\partial Q}{\partial {\mathbf {z} }^{\left( l\right) }_{b} } \\ \frac{\partial Q}{\partial \mathbf {c}^{\left( l\right) } }&=\frac{\partial Q}{\partial {\mathbf {z} }^{\left( l\right) }_{r} } \frac{\partial {\mathbf {z} }^{\left( l\right) }_{r} }{\partial \mathbf {c}^{\left( l\right) } } =\sum \frac{\partial Q}{\partial {\mathbf {z} }^{\left( l\right) }_{b} } \end{aligned} \end{aligned}$$

and

14 $$\begin{aligned} \begin{aligned} \frac{\partial Q}{\partial \mathbf {a}^{\left( l\right) } }&=\frac{\partial Q}{\partial \mathbf {z}^{\left( l\right) }_{r} } \frac{\partial \mathbf {z}^{\left( l\right) }_{r} }{\partial \mathbf {a}^{\left( l\right) } } +\frac{\partial Q}{\partial \mathbf {z}^{\left( l\right) }_{g} } \frac{\partial \mathbf {z}^{\left( l\right) }_{g} }{\partial \mathbf {a}^{\left( l\right) } } +\frac{\partial Q}{\partial \mathbf {z}^{\left( l\right) }_{b} } \frac{\partial \mathbf {z}^{\left( l\right) }_{b} }{\partial \mathbf {a}^{\left( l\right) } }\\&=\frac{\partial Q}{\partial \mathbf {z}^{\left( l\right) }_{r} } \left( \mathbf {w}^{\left( l\right) }_{r} \right) ^{T} +\frac{\partial Q}{\partial \mathbf {z}^{\left( l\right) }_{g} } \left( \mathbf {w}^{\left( l\right) }_{g} \right) ^{T}\\&\qquad +2\mathbf {a}^{\left( l\right) } \left[ \frac{\partial Q}{\partial \mathbf {z}^{\left( l\right) }_{b} } \left( \mathbf {w}^{\left( l\right) }_{b} \right) ^{T} \right] \end{aligned} \end{aligned}$$

In contrast to the forward computation, the input to the BP procedure is the predicted result $\mathbf {y}$, which is the output of the forward process. For layer $l = L, \dots , 1$, one layer at a time, we compute $\partial Q/\partial \mathbf {z}^{\left( l\right) }$ using Eqs. 9 or 10 depending on whether it is the last layer, the same for the conventional and quadratic neural networks. Then, we compute $\partial Q/\partial \theta ^{(l)}$ according to Eqs. 11 (for conventional neurons) or 13 (for quadratic neurons) respectively, where $\theta$ denotes a vector of all trainable parameters of the network. Finally, we compute $\partial Q/\partial \mathbf {a}^{\left( l\right) }$, which is used in Eqs. 12 (for conventional neurons) and 14 (for quadratic neurons) respectively for the next iteration. After the gradient of the network is obtained, we update the parameters via 'Adam' in this study.

#### Whole training process

**Initiation.** Let us use a series of integers to describe a feed-forward neural network architecture of our interest,

15 $$\begin{aligned} \mathbf {d} = d^{\left( 1\right) },\cdots ,d^{\left( l\right) },\cdots ,d^{\left( L+1\right) } \end{aligned}$$

where $d^{\left( l\right) }$ represents the dimension of $\mathbf {a}^{(l)}$. Then, the total number of neurons used in the network is $\sum \nolimits ^{L}_{l=1} d^{\left( l+1\right) }$. Note that $d^{\left( 1\right) } = D$, the dimension of input samples, and $d^{\left( L+1\right) } = K$, the number of classes.

Then, the network can be randomly initialized with a vector of parameters $\theta ^{\left( l\right) }$ for each layer. Specifically, for each layer $l\in [1, L]$, let d_from be the input dimension $d^{\left( l\right) }$ and d_to the output dimension $d^{\left( l+1\right) }$ Setting all weights – $\mathbf {w}^{(l)}$ for a conventional neural network and $\mathbf {w}_r^{(l)}, \mathbf {w}_g^{(l)}, \mathbf {w}_b^{(l)}$ for a quadratic neural network – and biases – $\mathbf {b}^{(l)}$ for a conventional network and $\mathbf {b}_r^{(l)}, \mathbf {b}_g^{(l)}, \mathbf {c}^{(l)}$ for a quadratic network – as follows:

$$\begin{aligned} \begin{aligned} \text{weight}&= 0.01 \cdot \mathtt{np.random.randn(d\_from, d\_to)}\\ \text{bias}&= \mathtt{np.zeros([1, d\_to])} \end{aligned} \end{aligned}$$

where np stands for NumPy (version 1.23.0), a Python package. That is, the bias is a 1 by $d^{\left( l+1\right) }$ zero matrix, and the weight is a $d^{\left( l\right) }$ by $d^{\left( l+1\right) }$ matrix.

**Optimization.** As shown in Fig. 1, given a neural network we just initialized and a training dataset containing samples $\mathbf {x}$ including the corresponding labels $\mathbf {t}$, we can repeat the forward computation and BP processes described in the above two subsections until the stopping criteria are satisfied. The cross-entropy losses on the training and validation samples will be estimated during the training process.

## Results and discussion

Using the training methods in the preceding section, we optimized conventional and quadratic neural networks to solve a number of GMM-based classification problems. At the beginning, we solved a three-class problem in the two-dimensional (2D) space to illustrate the working principle. Then, we performed a systematic comparison between conventional and quadratic neural networks on samples with different numbers of classes and dimensions. Finally, we applied all methods on three real data sets. Meanwhile, we used the EM algorithm and Bayes inference to obtain the Bayes error rate as the performance benchmark of the neural networks.

### Illustrative classification example

**Fig. 5:**
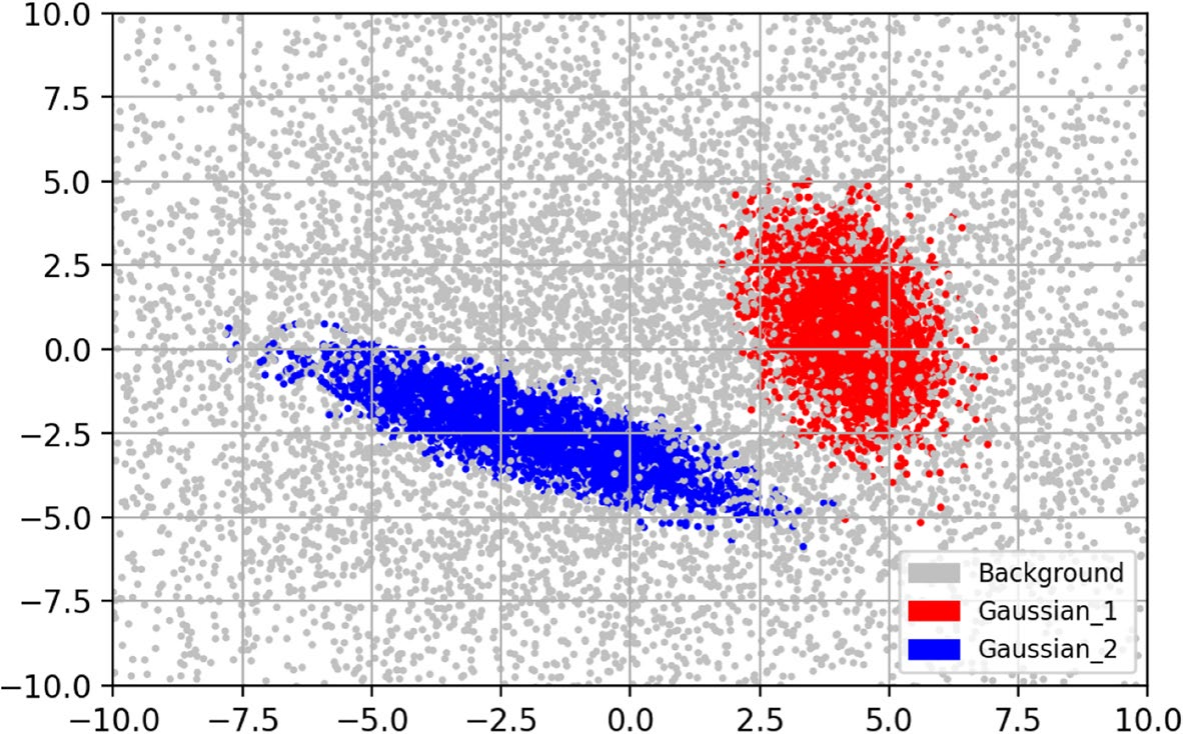
Scatter plot of sample points which contains three classes: two Gaussian clusters plus a background

Our initial classification problem assumes a finite number of classes (the first example, *K* = 3) in the 2D space ($D = 2$): two Gaussian clusters plus a background, which can be viewed as a special case of the Gaussian distribution. As in other network-based classification networks, a one-hot vector was used in our networks as well. The parameters of the background were set to

$$\begin{aligned} \mu _{b} = \left[ \begin{array}{c} 0\\ 0 \end{array}\right] , \mathbf {C}_{b} = \left[ \begin{array}{cc} 40&{}0\\ 0&{}40 \end{array}\right] \end{aligned}$$

where *b* indicates the background. Then, we randomly set the parameters of the other Gaussian clusters as

$$\begin{aligned} \begin{aligned} \mu _{k}&= \mathtt{(np.random.random(D) - 0.5) * 10}\\ a&=\mathtt{np.random.random((D, D)) * 2 - 1}\\ \mathbf {C}_{k}&=2aa^T \end{aligned} \end{aligned}$$

for $k = 1, \dots , K-1$ where $aa^T$ stands for matrix multiplication and np stands for NumPy (version 1.23.0), a Python package. Given the mean $\mu _{k}$ and covariance $\mathbf {C}_{k}$, we generated $N_k$ points for each class except the background where $N_k \in \left[ 20000, 30000\right]$ was chosen randomly. Then, we generated $N_{b}=\sum \nolimits ^{K-1}_{k=1} N_{k}$ points for the background. The entire dataset was shuffled and split into the three parts: 50% as training samples, 20% as validation samples, and 30% as test samples. Figure 5 shows the scatter plot of sample points.

**Fig. 6:**
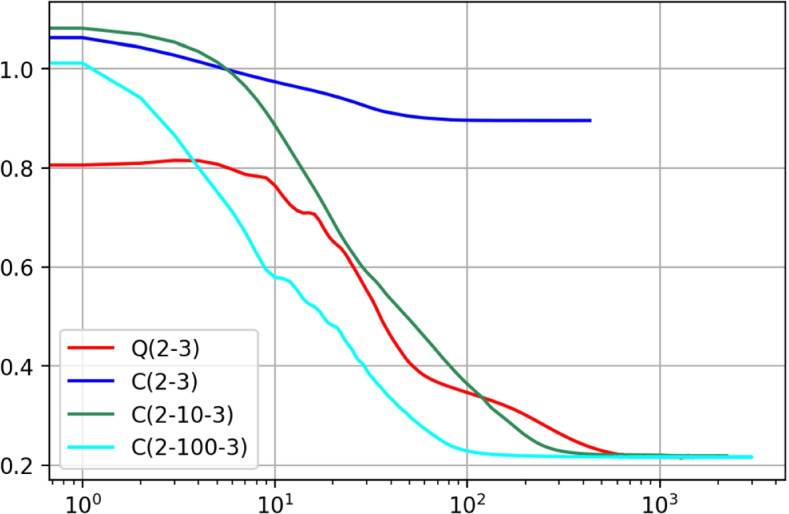
Decreasing loss on the validation samples during the training process of conventional (C) and quadratic (Q) neural networks with different numbers of neurons. The notation $(\cdot )$ stands for the architecture of a neural network as described in Eq. 15

**Fig. 7:**
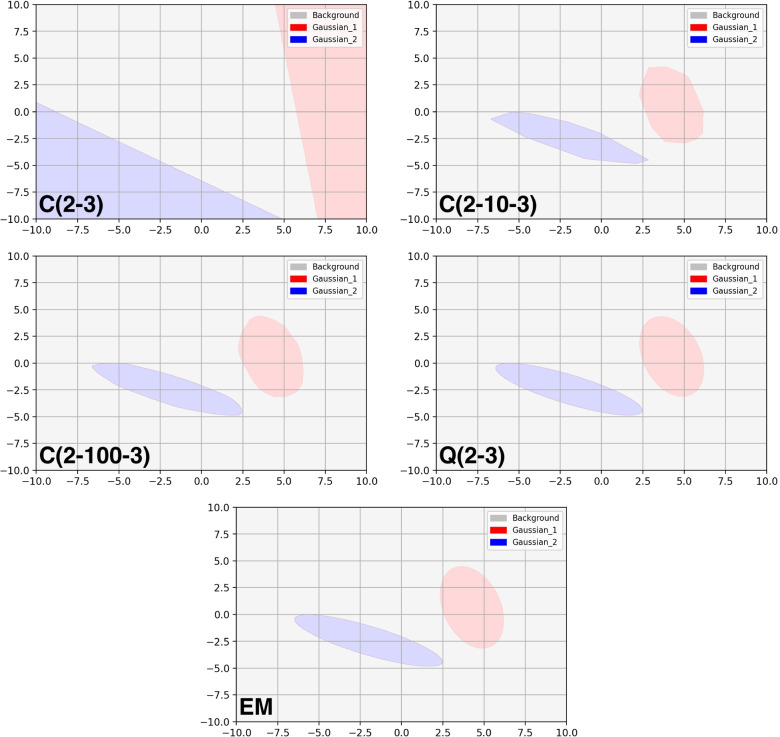
Decision boundaries of EM and neural networks, including C and Q neural networks with different numbers of neurons. The notation $(\cdot )$ stands for the architecture of a neural network as described in Eq. 15

We trained conventional and quadratic neural networks with different numbers of neurons for GMM classification. The decreasing loss is shown in Fig. 6 on the validation samples during the training process. The decision boundaries are shown in Fig. 7 for the conventional and quadratic neural networks as well as EM algorithm respectively. It took hundreds of neurons for the conventional network to approach the elliptical boundaries, while the quadratic network accurately fitted them with only three quadratic neurons.

The lighter the network structure, the higher the computation efficiency. Table 1 shows time spent to train the conventional and quadratic neural networks, and the accuracies of the EM and neural networks on the test samples. Our quadratic neural network with only one neural layer produced a performance closer to the EM benchmark than the conventional neural network of more than one hundred conventional neurons. Also, the time need for the quadratic neural network is only about 7% that of the conventional counterpart.

**Table 1:** The average accuracy of and time needed by EM algorithm, Q and C neural networks with different numbers of neurons in 2D spaces with two Gaussian clusters plus a background. The notation $(\cdot )$ stands for the architecture of a neural network as described in Eq. 15

	Accuracy (%)	Time (s)
C(2-3)	31.1752	2.44
C(2-10-3)	91.8122	23.15
C(2-100-3)	91.8391	213.61
Q(2-3)	91.8525	16.30
EM	91.8625	

### Systematic comparation

**Fig. 8:**
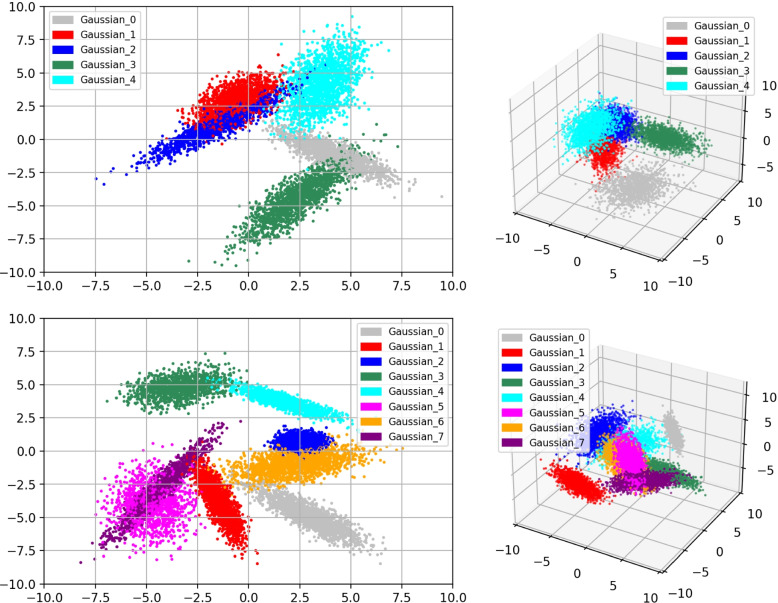
Typical scatter plots of samples in 2D (left) and 3D (right) spaces with $K=5$ (top) and $K=8$ (bottom)

To systematically compare conventional and quadratic networks, we tested conventional and quadratic networks in 2D and three-dimensional (3D) spaces with $K=5$ and 8 Gaussian clusters. In each case, we randomly generated 50 samples using the aforementioned method except we replaced the background by a Gaussian cluster and set $N_k \in \left[ 6000, 9000\right]$. Typical scatter plots of these samples are represented in Fig. 8.

We trained and tested the EM algorithm, conventional and quadratic networks with different numbers of layers/neurons in terms of the average accuracy. The resutls are summarized in Table 2. Very interestingly, in all cases, the accuracy of the quadratic networks with only output layer of few neurons is higher than that of the conventional network of over one hundred neurons. Meantime, the training time needed for quadratic neural networks is only about $26.82\%$, on average, of that taken by the much more complicated conventional network. Generally speaking, the quadratic neural networks delivered a performance very close to that of the EM algorithm.

**Table 2:** The average accuracy of and time needed by EM algorithm, Q and C neural networks with different numbers of neurons in 2D and 3D spaces with $K=5$ and 8 Gaussian clusters. The notation $(\cdot )$ stands for the architecture of a neural network as described in Eq. 15

	Accuracy (%)	Time (s)	Accuracy (%)	Time (s)
	D = 2, K = 5		D = 3, K = 5	
C(2-3)	$92.36 \pm 7.89$	$11.5 \pm 5.0$	$84.72 \pm 10.32$	$13.7 \pm 5.8$
C(2-10-3)	$95.36 \pm 5.66$	$23.3 \pm 14.1$	$92.15 \pm 8.04$	$24.8 \pm 8.6$
C(2-100-3)	$95.47 \pm 5.68$	$62.7 \pm 27.9$	$92.54 \pm 8.01$	$71.8 \pm 29.2$
Q(2-3)	$95.53 \pm 5.66$	$15.8 \pm 6.7$	$92.74 \pm 7.98$	$17.6 \pm 7.4$
EM	$95.60 \pm 5.65$		$92.90 \pm 7.97$	
	D = 2, K = 8		D = 3, K = 8	
C(2-3)	$83.84 \pm 5.53$	$22.8 \pm 8.0$	$76.47 \pm 6.31$	$28.8 \pm 10.6$
C(2-10-3)	$87.90 \pm 3.75$	$47.6 \pm 17.1$	$82.45 \pm 5.36$	$62.6 \pm 18.5$
C(2-100-3)	$88.06 \pm 3.70$	$122.6 \pm 39.2$	$82.68 \pm 5.34$	$151.8 \pm 41.4$
Q(2-3)	$88.13 \pm 3.67$	$33.3 \pm 11.0$	$82.79 \pm 5.31$	$46.2 \pm 13.3$
EM	$88.19 \pm 3.64$		$82.86 \pm 5.32$	

### Real data

**Fig. 9:**
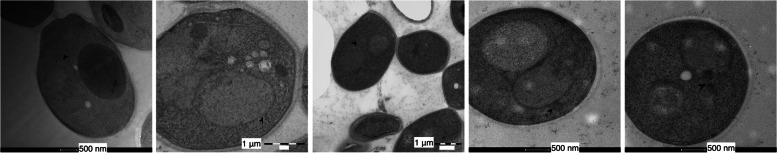
Typical yeast images from the Cell Image Library (http://cellimagelibrary.org/groups/50815)

Finally, we applied conventional and quadratic networks on three real data sets from the UCI Machine Learning repository [20]: protein localization sites (yeast), pen-based recognition of handwritten digits (pendigits), and isolated letter speech recognition (isolet). All three data sets’ attribute types are numerical. Some basic information about these datasets are in Table 3. For the yeast dataset, we split the whole dataset in the same proportions as that described in the first subection. Typical yeast cell (Saccharomyces cerevisiae cell) images the Cell Image Library [21] are shown in Fig. 9, visualized through transmission electron microscopy. For the pendigits and isolet datasets, with the test samples already provided, 30% of training samples were used for validation.

**Table 3:** Basic information about three real data sets: protein localization sites (yeast), pen-based recognition of handwritten digits (pendigits), and isolated letter speech recognition (isolet)

Datasets	Train	Test	Dimensions	Classes
yeast	1484		8	10
pendigits	7494	3498	16	10
isolet	6238	1559	617	26

We trained and tested the EM algorithm, conventional and quadratic networks with different numbers of layers/neurons on each dataset 20 times. The average accuracy of and time needed by each method are shown in Table 4. In each application, the quadratic neural network with only layer of few neurons has the highest accuracy while its training time is about half of the conventional networks orders of magnitude larger than the quadratic version.

**Table 4:** The average accuracy of and time needed by Q and C neural networks with different numbers of neurons for three real datasets. The notation $(\cdot )$ stands for the architecture of a neural network as described in Eq. 15

	Yeast		Pendigits		Isolet	
	Accuracy (%)	Time (s)	Accuracy (%)	Time (s)	Accuracy (%)	Time (s)
C(2-3)	$57.17 \pm 1.79$	$0.5 \pm 0.3$	$90.01 \pm 1.54$	$3.3 \pm 2.7$	$89.94 \pm 3.30$	$11.2 \pm 0.2$
C(2-10-3)	$58.21 \pm 1.93$	$0.7 \pm 0.2$	$93.23 \pm 2.39$	$4.0 \pm 1.9$	$92.00 \pm 0.60$	$11.8 \pm 0.7$
C(2-100-3)	$59.69 \pm 2.96$	$0.9 \pm 0.2$	$96.66 \pm 0.29$	$14.8 \pm 27.4$	$94.47 \pm 0.30$	$49.8 \pm 2.6$
Q(2-3)	$60.99 \pm 1.27$	$0.5 \pm 0.1$	$97.04 \pm 0.30$	$6.1 \pm 0.2$	$95.01 \pm 0.17$	$21.2 \pm 0.2$

## Conclusions

Although it has been well tested with a solid theoretical foundation, the EM algorithm needs to take an entire dataset into the memory, processes them iteratively, and is time-consuming, under the restriction that data must come from GMM. Furthermore, when new samples become available, parameters need to be adjusted again. A neural network approach can be much more desirable, effective and efficient, workable with many data models in principle thanks to its universal approximation nature. After a network is well trained, new samples can be used to fine-tune the network or processed to inference in a feed-forward fashion, being extremely efficient and generalizable to cases much more complicated than GMM. Very interestingly, compared to conventional networks, quadratic networks can deliver a performance close to that of the EM algorithm in the GMM cases and yet be orders of magnitude simpler than conventional networks for the same classification task.

In conclusion, in this paper we have numerically and experimentally demonstrated the superiority of quadratic networks over conventional ones. It is underlined that the quadratic neural network of a much lighter structure rivals the conventional network of a complexity orders of magnitude more in solving the same classification problems. Clearly, the superior classification performance of quadratic networks could be translated to medical imaging tasks, especially radiomics.

## Data Availability

The datasets analysed during the current study are available in the UCI Machine Learning repository, http://archive.ics.uci.edu [20]. Applications and source codes are available at https://github.com/tianrui-qi/QuadraticNeurons.

## Abbreviations

ANN: Artificial neural network
BP: Backpropagation
EM: Expectation-maximization
GMM: Gaussian mixture model
2D: Two-dimensional
3D: Three-dimensional
